# Supplementary material for: HIV status alters disease severity and immune cell responses in Beta variant SARS-CoV-2 infection wave
Source: eLife. 2021 Oct 5;10:e67397. doi: 10.7554/eLife.67397 (PMC8676326; doi:10.7554/eLife.67397)
Supplement: Supplementary file 1. [file elife-67397-supp1.docx]

Supplementary File 1: Summary of case visits

| Timepoint | Visit | Participants |
| --- | --- | --- |
| 1 | Enrollment | 236 (100.0) |
| 2 | DAY 7 | 157 (66.5) |
| 3 | DAY 14 | 139 (58.9) |
| 4 | DAY 21 | 125 (53.0) |
| 5 | DAY 28 | 142 (60.2) |
| 6 | MONTH 3 | 87 (36.9) |
| 7 | MONTH 6 | 72 (30.5) |
| 8 | MONTH 9 | 24 (10.2) |
| Unscheduled visits | - | 4 (1.7) |
| Total case visits | **-** | **986** |
