# Supplementary material for: HIV status alters disease severity and immune cell responses in Beta variant SARS-CoV-2 infection wave
Source: eLife. 2021 Oct 5;10:e67397. doi: 10.7554/eLife.67397 (PMC8676326; doi:10.7554/eLife.67397)
Supplement: Supplementary file 2. [file elife-67397-supp2.docx]

Supplementary File 2: Timing of enrollment in PLWH and HIV negative participants

|  |  | All Participants | HIV- | HIV+ |
| --- | --- | --- | --- | --- |
| Median (IQR) days symptom onset to enrollment | | 11 (8 – 18) | 11 (8 – 18) | 11 (8 – 18) |
| Median (IQR) days diagnostic swab to enrollment | | 8 (5 -14) | 8.5 (5 – 17.5) | 7.5 (5 – 10) |
| Median (IQR) days symptom onset to diagnostic swab | | 3 (1 – 6) | 3 (2 – 6) | 3 (1 – 7) |
