# Supplementary material for: HIV status alters disease severity and immune cell responses in Beta variant SARS-CoV-2 infection wave
Source: eLife. 2021 Oct 5;10:e67397. doi: 10.7554/eLife.67397 (PMC8676326; doi:10.7554/eLife.67397)
Supplement: Supplementary file 3. [file elife-67397-supp3.docx]

Supplementary File 3: ART regimen in PLWH as determined by LC-MS/MS

| LC-MS/MS determined Regimen | Number of participants (%) |
| --- | --- |
| EFV based regimen | 41 (44.1) |
| EFV regimen transitioning to DTG regimen | 15 (16.1) |
| NVP/AZT based regimen | 3 (3.2) |
| DTG based regimen | 4 (4.3) |
| LPV/r based regimen | 6 (6.5) |
| ATV based regimen | 3 (3.2) |
| Combination of TFV, FTC, or 3TC only | 5 (5.4) |
| No detectible ART yet record of ART regimen | 5 (5.4) |
| ART naïve | 6 (6.5) |
| Information not available | 5 (5.4) |
