# Supplementary material for: HIV status alters disease severity and immune cell responses in Beta variant SARS-CoV-2 infection wave
Source: eLife. 2021 Oct 5;10:e67397. doi: 10.7554/eLife.67397 (PMC8676326; doi:10.7554/eLife.67397)
Supplement: Supplementary file 4. [file elife-67397-supp4.docx]

Supplementary File 4: Infection wave 1 COVID-19 disease severity by HIV status

|  | All  (n=153) | HIV-  (n= 90, 58.8%) | HIV+  (n=63, 41.2%) | Odds Ratio  (95% CI) | p-value^#^ |
| --- | --- | --- | --- | --- | --- |
| Asymptomatic | 25 (16.3) | 18 (20.0) | 7 (11.1) | 0.5 (0.2 – 1.3) | 0.18 |
| Ambulatory with symptoms | 89 (58.2) | 51 (56.7) | 38 (60.3) | 1.2 (0.6 – 2.2) | 0.74 |
| Supplemental oxygen | 32 (20.9) | 17 (18.9) | 15 (23.8) | 1.3 (0.6 – 2.9) | 0.54 |
| Death | 7 (4.6) | 4 (4.4) | 3 (4.8) | 1.1 (0.3 – 4.5) | >0.99 |

**^#^** p-value calculated via 2-sided Fisher’s Exact test.
