# Supplementary material for: HIV status alters disease severity and immune cell responses in Beta variant SARS-CoV-2 infection wave
Source: eLife. 2021 Oct 5;10:e67397. doi: 10.7554/eLife.67397 (PMC8676326; doi:10.7554/eLife.67397)
Supplement: Supplementary file 5. [file elife-67397-supp5.docx]

Supplementary File 5: Infection wave 2 COVID-19 disease severity by HIV status

|  | All  (n=83) | HIV-  (n= 53, 63.9%) | HIV+  (n=30, 36.1%) | Odds Ratio  (95% CI) | p-value^#^ |
| --- | --- | --- | --- | --- | --- |
| Asymptomatic | 8 (9.6) | 7 (13.2) | 1 (3.3) | 0.2 (<0.1 – 1.5) | 0.25 |
| Ambulatory with symptoms | 39 (47.0) | 29 (54.7) | 10 (33.3) | 0.4 (0.2 – 1.0) | 0.071 |
| Supplemental oxygen | 30 (36.1) | 13 (24.5) | 17 (56.7) | **4.0 (1.6 – 10.4)** | **0.005** |
| Death | 6 (7.2) | 4 (7.5) | 2 (6.7) | 0.9 (<0.1 – 4.4) | >0.99 |

**^#^** p-value calculated via 2-sided Fisher’s Exact test.
