# Supplementary material for: HIV status alters disease severity and immune cell responses in Beta variant SARS-CoV-2 infection wave
Source: eLife. 2021 Oct 5;10:e67397. doi: 10.7554/eLife.67397 (PMC8676326; doi:10.7554/eLife.67397)
Supplement: Supplementary file 6. [file elife-67397-supp6.docx]

Supplementary File 6: Comparison between HIV negative participants requiring and not requiring supplemental oxygen

|  | All  (n=143) | No Supp. O_2_  (n=108, 75.5%) | Supp. O_2_  (n=35, 24.5%) | Odds Ratio  (95% CI) | p-value^#^ |
| --- | --- | --- | --- | --- | --- |
| Demographics |  |  |  |  |  |
| Age years, median (IQR) | 49 (35-62) | 46.5 (34-57) | 62 (47-66) | - | 0.002* |
| Comorbidity, n (%) |  |  |  |  |  |
| Hypertension^$^, n= 142 | 42 (29.4) | 24 (22.2) | 18 (51.4) | 3.7 (1.7 – 8.1) | 0.002 |
| Diabetes | 32 (22.4) | 19 (17.6) | 13 (37.1) | 2.8 (1.2 – 6.4) | 0.021 |
| Obesity^$^, n= 136 | 64 (47.1) | 53 (40.1) | 11 (31.4) | 0.5 (0.2 – 1.1) | 0.11 |
| Active TB | 1 (0.7) | 0 (0.0) | 1 (2.9) | <0.1 (0 – >10) | 0.25 |
| History TB | 3 (2.1) | 1 (0.9) | 2 (5.7) | 6.5 (0.8 – >10) | 0.15 |
| COVID-19 treatment, n (%) |  |  |  |  |  |
| Corticosteroids | 47 (32.9) | 22 (20.4) | 25 (71.4) | 9.8 (4.1 – >10) | <0.0001 |
| Anticoagulants | 35 (24.5) | 17 (15.7) | 18 (51.4) | 5.7 (2.5 – >10) | 0.0001 |

**^#^** p-value calculated via 2-sided Fisher’s Exact test, except for * which was calculated via Mann-Whitney U test. ^$^Not including pregnancy or unable to be measured.
