# Supplementary material for: HIV status alters disease severity and immune cell responses in Beta variant SARS-CoV-2 infection wave
Source: eLife. 2021 Oct 5;10:e67397. doi: 10.7554/eLife.67397 (PMC8676326; doi:10.7554/eLife.67397)
Supplement: Supplementary file 7. [file elife-67397-supp7.docx]

Supplementary File 7: Comparison between PLWH requiring and not requiring supplemental oxygen

|  | All  (n=93) | No Supp.O_2_  (n= 60, 64.5%) | Supp.O_2_  (n=33, 35.5%) | Odds Ratio  (95% CI) | p-value^#^ |
| --- | --- | --- | --- | --- | --- |
| Demographic characteristic |  |  |  |  |  |
| Age years, median (IQR) | 41 (35-50) | 40.5 (34-49) | 41 (36-56) | - | 0.295* |
| Comorbidity, n (%) |  |  |  |  |  |
| Hypertension | 15 (16.1) | 7 (11.7) | 8 (24.2) | 2.4 (0.8 – 7.2) | 0.144 |
| Diabetes | 10 (10.8) | 6 (10.0) | 4 (12.1) | 1.2 (0.3 – 4.5) | 0.739 |
| Obesity^$^, n= 79 | 27 (34.2) | 15 (25.0) | 12 (36.4) | 2.4 (0.9 – 6.4) | 0.125 |
| Active TB | 9 (9.7) | 4 (6.7) | 5 (15.2) | 2.5 (0.7 – 9.3) | 0.272 |
| History TB | 29 (31.2) | 15 (25.0) | 14 (42.4) | 2.2 (0.9 – 5.4) | 0.103 |
| COVID-19 treatment, n (%) |  |  |  |  |  |
| Corticosteroids | 27 (29.0) | 9 (15.0) | 18 (54.5) | 6.8 (2.6 – >10) | 0.0001 |
| Anticoagulants | 18 (19.4) | 5 (8.3) | 13 (39.4) | 7.2 (2.3 – >10) | 0.001 |

**^#^** p-value calculated by 2-sided Fisher’s Exact test, except for * which was calculated via Mann-Whitney U test. ^$^Not including pregnancy or unable to measure.
